# Real-world pharmacovigilance of drug-related bone metabolism disorders: integrating FAERS and VigiAccess with a Bradford Hill-based causal plausibility assessment

**DOI:** 10.3389/fphar.2026.1766498

**Published:** 2026-06-19

**Authors:** Pei Liu, Kun Zhao, Hong Zhang, Ming Qiang Liu, Jianlei Li, Yongqiang Sun

**Affiliations:** 1 Department of Artificial Joint Revision, Henan Luoyang Orthopedic Hospital (Henan Provincial Orthopedic Hospital), Zhengzhou, Henan, China; 2 Henan Provincial Engineering Research Center for Traditional Chinese Medicine Prevention and Treatment of Bone Tumors, Zhengzhou, Henan, China; 3 Shanghai Jiao Tong University Affiliated Sixth People’s Hospital South Campus, Shanghai, China

**Keywords:** disproportionality analysis, drug safety, FAERS, metabolic bone disease, osteoporosis, pharmacovigilance, signal detection, VigiAccess

## Abstract

**Background:**

Metabolic bone diseases (MBDs) are a group of conditions characterized by imbalanced bone remodeling, including osteoporosis, osteomalacia, and rickets. In recent years, drug-induced secondary MBDs have emerged as a major clinical concern. However, systematic drug risk assessment studies based on large-scale real-world data remain scarce.

**Methods:**

Based on the U.S. Food and Drug Administration Adverse Event Reporting System (FAERS, 2004–2024) and VigiAccess, four complementary disproportionality algorithms (reporting odds ratio, ROR; proportional reporting ratio, PRR; Bayesian confidence propagation neural network, BCPNN; multi-item gamma Poisson shrinker, MGPS) were used to screen drug-event signals. The signals were stratified by gender and age type, and their temporal distribution characteristics were characterized using the Weibull model. Finally, the causal plausibility was assessed using the updated Bradford Hill framework.

**Results:**

A total of 55,783 adverse event reports were included, and 33 drugs showed consistent positive signals in both the FAERS and VigiAccess databases. Major signal drugs included bisphosphonates (e.g., alendronic acid, zoledronic acid), antiviral drugs (e.g., tenofovir disoproxil fumarate, emtricitabine/tenofovir combination), proton pump inhibitors (e.g., esomeprazole), and endocrine antineoplastic drugs (e.g., anastrozole). Within the Anatomical Therapeutic Chemical (ATC) classification, signals were primarily concentrated in anti-infectives (category J), antineoplastic and immunomodulatory drugs (category L), and musculoskeletal drugs (category M).

**Conclusion:**

This study systematically identified robust pharmacovigilance signals and disproportionality-based associations between multiple drug classes and metabolic bone disorders through an integrated analysis of the FAERS and VigiAccess databases, providing high-confidence real-world evidence for pharmacovigilance signal detection.

## 1 Introduction

Metabolic bone diseases (MBDs) are disorders caused by disrupted bone remodeling, leading to reduced bone mass, microarchitectural deterioration, and increased fragility. With population aging, the incidence of MBDs has increased significantly, and these disorders have become an important cause of fractures, disability, and reduced quality of life in older adults. In addition to primary causes, drug-induced secondary metabolic bone disorders are gradually becoming a clinical problem that cannot be ignored. Glucocorticoid-induced osteoporosis is currently the most common drug-induced MBD in clinical practice. Long-term or high-dose glucocorticoid use can inhibit osteoblast activity, promote bone resorption, and disrupt calcium and phosphorus metabolism, ultimately leading to decreased bone density (Hofbauer et al., 2025; Chen et al., 2023). Agents including aromatase inhibitors, enzyme-inducing antiepileptics (e.g., phenytoin), and proton-pump inhibitors have been consistently linked to disturbances in bone metabolism (Lespessailles and Toumi, 2022). Studies have shown that aromatase inhibitors can disrupt bone remodeling and induce osteoporosis by reducing estrogen levels (Xu et al., 2023; Hadji et al., 2025). Traditional antiepileptic drugs such as phenytoin, phenobarbital, and carbamazepine induce cytochrome P450 (CYP450) isoenzyme activity, inhibit vitamin D synthesis, cause hypocalcemia, and significantly reduce bone mineral density (BMD) (Gaete et al., 2025; Mikati and El-Hajj Fuleihan, 2023). Furthermore, antiviral drugs such as tenofovir can not only lead to decreased BMD but also cause serious complications such as hypophosphatemic osteomalacia and Fanconi syndrome, which have attracted widespread concern regarding drug safety (Hadzimuratovic et al., 2021; Joseph et al., 2023; Sahoo and Wakhlu, 2022; Mateo et al., 2016). Pharmacovigilance databases, as important tools for monitoring adverse drug reactions (ADRs), can identify rare or delayed drug risks based on real-world data. Among them, the U.S. Food and Drug Administration Adverse Event Reporting System (FAERS) is the largest publicly available spontaneous-report repository and offers near-real-time signal detection capability (Dhodapkar et al., 2022), while the World Health Organization global pharmacovigilance database, VigiAccess, provides supplementary evidence across countries and populations, allowing researchers to verify the robustness and consistency of drug risk signals on a global scale (Shankar, 2016).

Based on the two global pharmacovigilance databases, FAERS and VigiAccess, this study systematically identified drug risk signals associated with MBDs. By integrating four disproportionality algorithms (reporting odds ratio, ROR; proportional reporting ratio, PRR; Bayesian confidence propagation neural network, BCPNN; and multi-item gamma Poisson shrinker, MGPS) with a modified Bradford-Hill-based causal plausibility framework, we performed cross-database comparisons and signal integration analyses across different drug classes. We aimed to characterize the pharmacovigilance signal landscape of drug-associated metabolic bone disorders and to generate evidence for risk-stratified safety monitoring.

## 2 Methods

### 2.1 Data sources and processing

Data for this study were obtained from FAERS and the World Health Organization Global Case Safety Reporting Database (VigiAccess). The original FAERS ASCII files were downloaded from the FDA website and imported into SAS 9.4, where they were cleaned according to the FDA’s latest deduplication guidelines. First, the records were sorted in ascending order by CASEID, FDA_DT, and PRIMARYID. For records with the same CASEID, the entry with the most recent FDA_DT and the largest PRIMARYID was retained. Subsequently, duplicate reports listed in the FDA 2019 deletion list were removed. Drug substances were mapped to the WHO Drug Dictionary (version September 2024) to ensure semantic consistency across reports.

The VigiAccess query was conducted on 31 December 2024. Publicly available aggregated case counts were retrieved for each target drug and each MBD-related event term using the same event definitions applied in the FAERS analysis. VigiAccess, maintained by the Uppsala Monitoring Centre, is a public access portal that provides statistical summaries of reports included in VigiBase, the WHO global database of individual case safety reports. Because VigiAccess provides aggregated rather than individual-level data, it does not support detailed case-level demographic analyses, but it can be used for cross-database disproportionality assessment. Drug-event counts and the corresponding comparator counts from VigiAccess were organized into 2 × 2 contingency tables using the same MedDRA-based event definitions as in FAERS, and the same signal detection framework was applied for cross-database validation.

### 2.2 Data screening and case definition

After cleaning and standardization, a total of 18,613,992 unique patient reports, representing 55,357,463 adverse event (AE) records, were obtained. Subsequently, events related to MBDs were screened using Preferred Term (PT) entries from MedDRA version 27.1, including osteoporosis, osteomalacia, bone demineralization, increased bone resorption, osteodystrophy, and bone lesions (Supplementary Table S1). A total of 55,783 MBD-related AE reports were included for subsequent analysis. For drug-event pairings, only cases in which the target drug was reported as the “primary suspect” were included to improve the specificity of drug-event signal detection.

### 2.3 Descriptive analysis

Descriptive statistics were used to characterize the overall features of adverse drug event (ADE) reports related to MBDs, including sex, age, reporting country, reporter type, severity, outcome, and reporting year. Event severity was categorized according to the FDA definition as life-threatening, hospitalization, disability, death, congenital anomaly, intervention required to prevent permanent injury, and other serious medical events. Because the public version of VigiAccess does not provide detailed demographic information at the individual level, descriptive analyses by demographic characteristics were limited to FAERS.

### 2.4 Disproportionality analysis

This study employed four classical signal detection algorithms: ROR, PRR, BCPNN, and MGPS. All analyses were performed using 2 × 2 contingency tables. Positive signal thresholds were defined as follows: ROR ≥3 with the lower bound of the 95% confidence interval >1; PRR ≥3 with chi-square ≥4; IC025 >0; and EBGM05 >2. A signal positive across all four algorithms was defined as a high-confidence signal, and cross-database validation was performed using FAERS and VigiAccess. Signals with positive results in both databases were retained as consistent signals (van Puijenbroek et al., 2002; Hauben et al., 2007; Bate et al., 1998). In disproportionality analysis, higher ROR or PRR values generally indicate more pronounced reporting disproportionality for a given drug-event pair. However, these frequentist measures may be influenced by reporting behavior, indication bias, competition bias, and concomitant medications. In contrast, Bayesian shrinkage metrics, including the information component (IC025) from BCPNN and the empirical Bayes geometric mean (EBGM05) from MGPS, generally provide more conservative and robust estimates, particularly for sparse data or rare events, by shrinking unstable estimates toward the null. Because these measures arise from different statistical frameworks, their numerical magnitudes are not directly comparable. Therefore, signal strength was interpreted comparatively and in conjunction with cross-method consistency, temporality, clinical specificity, and biological plausibility.

### 2.5 Subgroup analysis

To further explore risk differences among different populations, subgroup analyses were conducted based on the FAERS data. Gender-stratified analyses compared differences in ADE reporting between male and female patients. Age-stratified analyses were categorized into children and adolescents (<18 years), young adults (18–44 years), middle-aged adults (45–64 years), and elderly patients (≥65 years). Population-specific risk patterns were assessed by comparing the distribution and intensity of positive signal drugs across groups.

### 2.6 Time-to-onset (TTO) analysis

To delineate the temporal characteristics of AEs associated with MBD therapies, we extracted the interval between drug initiation (START_DT) and AE onset (EVENT_DT) from FAERS and fitted a two-parameter Weibull model. The scale (α) and shape (β) parameters jointly characterize the time-varying hazard. β < 1 with the 95% CI upper bound <1 indicates a decreasing hazard (early failure); β >1 with the 95% CI lower bound >1 indicates an increasing hazard (wear-out); β ≈ 1 with the 95% CI spanning 1 implies a constant hazard (random failure). Cumulative distribution curves were additionally generated to visualize the cumulative probability of AE occurrence across drugs (Leroy et al., 2014).

### 2.7 Evidence integration and causal plausibility assessment

Based on the signal detection results, this study comprehensively evaluated the causal plausibility of associations between drugs and MBDs from multiple dimensions with reference to the modified Bradford-Hill criterion (Suzuki and Yamamoto, 2021). The Bradford-Hill framework was applied as a qualitative approach to assess causal plausibility based on pharmacovigilance signals rather than to establish causality. Assessment criteria included signal strength (magnitude across four algorithms), cross-database consistency (FAERS vs. VigiAccess), phenotypic specificity (characteristics across different MBD subtypes), temporality (TTO/Weibull pattern), biological plausibility (pharmacological mechanism support), and comparability (risk comparison with similar drugs). Due to the inherent limitations of the spontaneous reporting system, dechallenge and rechallenge evidence were presented descriptively only and were not included in the causal plausibility assessment.

### 2.8 Statistical analysis

Categorical variables are expressed as counts and proportions, whereas continuous variables are presented as medians with interquartile ranges. Differences in TTO values across groups were examined using the Kruskal-Wallis test, with α = 0.05. Data processing and all statistical analyses were conducted using SAS 9.4 and R 4.4.2.

## 3 Results

### 3.1 Overall characteristics of reports

A total of 55,783 AE reports related to MBD were identified in the FAERS database (Supplementary Table S2). Reporters were predominantly female (34,857 cases, 62.49%), whereas 16,912 cases (30.32%) were male. Cases primarily originated from the United States (37,598 cases, 67.40%), followed by Canada (4,217 cases, 7.56%) and Germany (1,344 cases, 2.41%). Serious AEs accounted for 86.96% of all reports, including life-threatening events (1.90%), hospitalizations (29.38%), disability (10.05%), deaths (3.84%), congenital anomalies (0.40%), interventions required to prevent permanent damage (0.33%), and other medically significant events (77.64%). The VigiAccess database retrieved 91,524 global reports related to MBD, indicating that drug-associated metabolic bone events have been reported across populations in multiple countries.

### 3.2 Signal detection results and high-confidence drug identification

**FIGURE 1:**
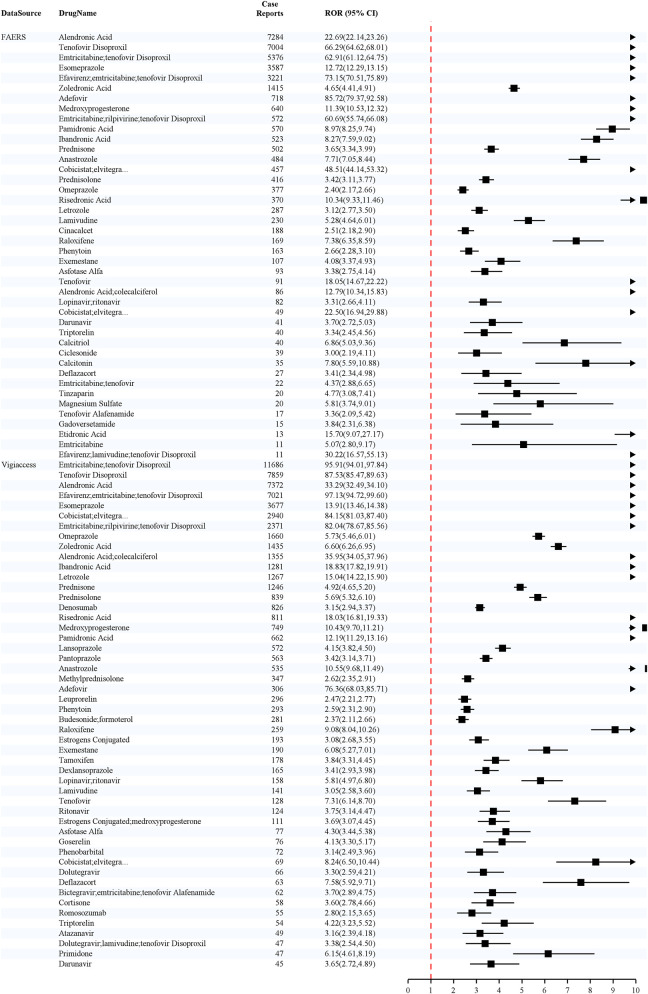
Distribution of the top 50 positive drug–event signals related to metabolic bone diseases detected by four disproportionality algorithms (ROR, PRR, IC, and MGPS) in both FAERS and VigiAccess databases, ranked by report frequency.

In the FAERS database, 42 drugs met the positive criteria after calculation using four disproportionality algorithms (ROR, PRR, BCPNN, and MGPS). The drugs with the strongest signals included alendronate (ROR = 22.69), tenofovir disoproxil fumarate (TDF, ROR = 66.29), emtricitabine/TDF (ROR = 62.91), esomeprazole (ROR = 12.72), and efavirenz/emtricitabine/TDF (ROR = 73.15) (Supplementary Table S3; Figure 1). In the VigiAccess database, analysis using the same four disproportionality algorithms (ROR, PRR, BCPNN, and MGPS) identified 97 positive drug signals associated with metabolic bone disease. The drugs with the strongest signals included emtricitabine/TDF (ROR = 95.91), TDF (ROR = 87.53), alendronate (ROR = 33.29), efavirenz/emtricitabine/TDF (ROR = 97.13), and esomeprazole (ROR = 13.91) (Supplementary Table S4; Figure 1). Drug ATC classification analysis in both databases showed that signals were concentrated in systemic anti-infectives (Class J), antitumor and immunomodulatory drugs (Class L), and musculoskeletal drugs (Class M), followed by digestive and metabolic drugs (Class A) and nervous system drugs (Class N) (Supplementary Tables S5, S6).

### 3.3 Subgroup analysis

**FIGURE 2:**
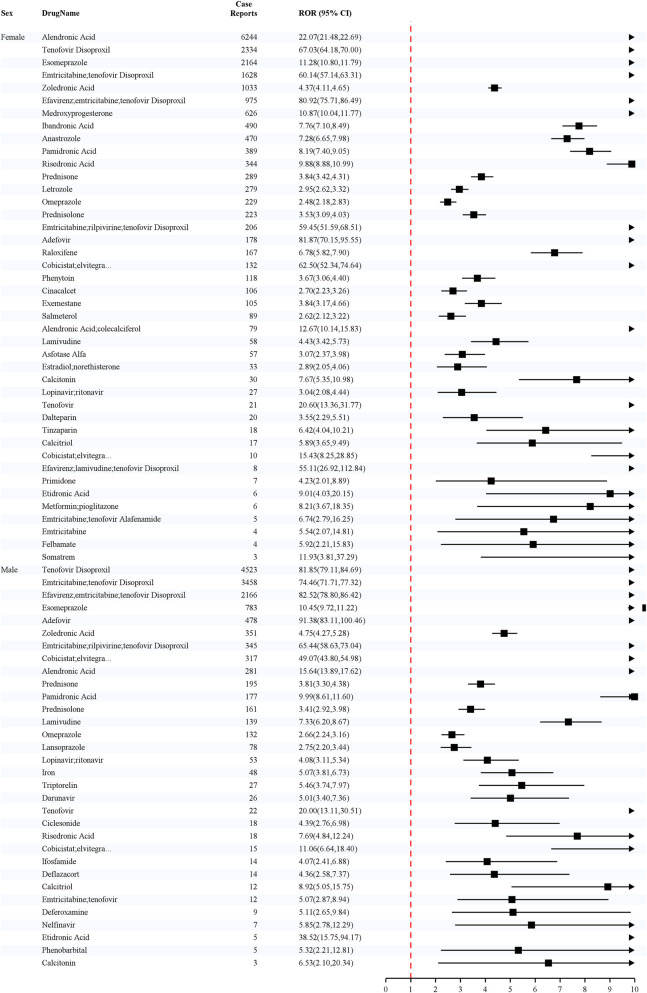
Distribution of the top 50 drug–event signals for metabolic bone diseases detected by four disproportionality algorithms, stratified by sex (FAERS database).

**FIGURE 3:**
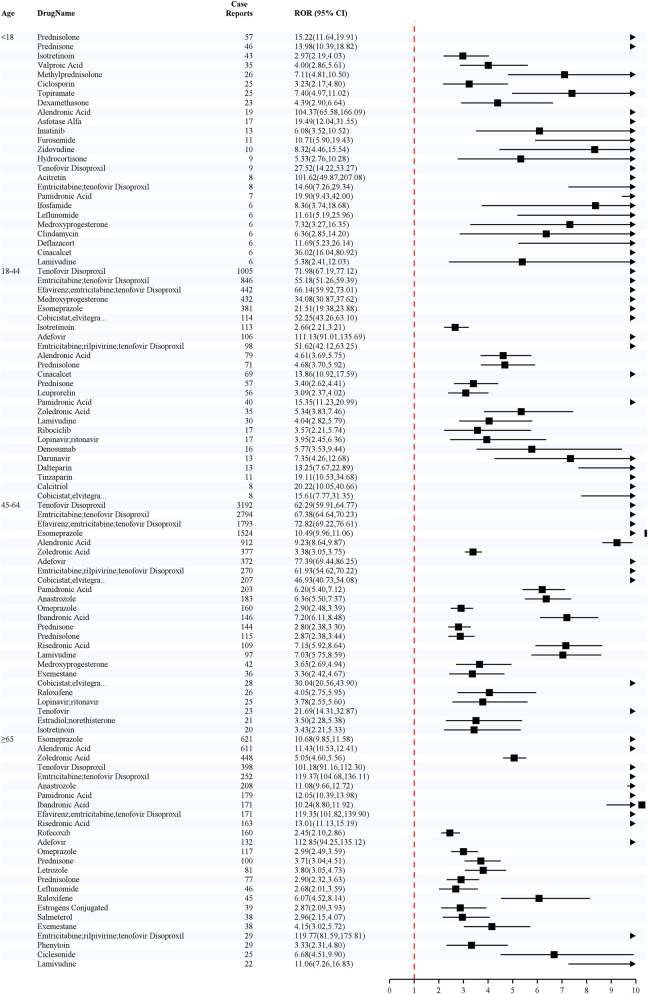
Distribution of the top 25 drug–event signals for metabolic bone diseases detected by four disproportionality algorithms, stratified by age group (FAERS database).

Sex-stratified analysis showed that AEs related to MBD in women were primarily associated with alendronate, tenofovir disoproxil, esomeprazole, emtricitabine/tenofovir disoproxil, and zoledronic acid, whereas in men they were primarily associated with tenofovir disoproxil, emtricitabine/tenofovir disoproxil, efavirenz/emtricitabine/tenofovir disoproxil, esomeprazole, and adefovir (Figure 2). Age-stratified analysis showed that in individuals under 18 years of age, most AEs were related to glucocorticoids and retinoids. Tenofovir and its combination preparations were the most prominent agents in the 18–44 age group. Tenofovir, alendronate, zoledronic acid, and adefovir were primarily reported in the 45–64 age group. Esomeprazole, alendronate, zoledronic acid, and anastrozole were the primary agents reported in individuals ≥65 years of age (Figure 3).

### 3.4 TTO and weibull distribution analysis

TTO analysis based on FAERS data showed significant differences in the time to MBD events among different medications (Supplementary Table S7). The overall median TTO was 577 days (IQR: 98–1737 days). Weibull distribution analysis revealed that the upper bound of the shape parameter (β) for most medications was less than 1, suggesting an early-failure pattern in which reported events may occur more frequently during the early treatment period and then decrease over time. Among these drugs, alendronate (β = 0.83) and zoledronic acid (β = 0.86) showed a typical early-failure pattern. In contrast, TDF (β = 1.27), emtricitabine/TDF (β = 1.26), efavirenz/emtricitabine/TDF (β = 1.40), adefovir (β = 1.44), medroxyprogesterone acetate (β = 2.05), and esomeprazole (β = 1.39) showed a wear-out failure pattern, suggesting that reported events may become more frequent with longer treatment duration. Some drugs, including anastrozole, pamidronate, ibandronate, and risedronate, had β values close to 1, consistent with a random-failure pattern in which reported events may occur throughout treatment. Notably, calcitriol (β = 11.94) showed a markedly elevated shape parameter, suggesting a possible late-onset reporting pattern. However, this estimate should be interpreted cautiously, particularly if it is based on a limited number of reports.

### 3.5 Cross-database signal integration

Combining the analysis results from FAERS and VigiAccess, a total of 33 drugs that showed positive results in both databases were identified as the final high-confidence signal set (Table 1). Their ROR values ranged from 2.40 to 85.72, with substantial variation in signal strength. The drugs with the strongest signals were adefovir, efavirenz/emtricitabine/TDF, and TDF. ATC classification results showed that systemic anti-infective drugs (Class J) ranked first in both signal quantity and signal strength, followed by musculoskeletal drugs (Class M) and antitumor and immunomodulatory drugs (Class L). This suggests that nucleos(t)ide analogues, bisphosphonates, and aromatase inhibitors exhibited the most consistent pharmacovigilance signals for MBD across both databases (Table 2).

**TABLE 1:** Cross-validated drug–event signals for metabolic bone diseases.

Drug name	Case	ROR (95% CI)	PRR (95% CI)	Chi-square	IC (IC025)	EBGM (EBGM05)
Alendronic acid	7,284	22.69 (22.14, 23.26)	22.21 (21.68, 22.75)	130,575	4.30 (4.26)	19.75 (19.27)
Tenofovir disoproxil	7,004	66.29 (64.62, 68.01)	62.18 (60.70, 63.70)	375,121	5.79 (5.74)	55.37 (53.97)
Emtricitabine; tenofovir disoproxil	5,376	62.91 (61.12, 64.75)	59.10 (57.52, 60.73)	281,144	5.76 (5.70)	54.14 (52.60)
Esomeprazole	3,587	12.72 (12.29, 13.15)	12.56 (12.14, 12.99)	36,021.3	3.57 (3.52)	11.90 (11.50)
Efavirenz; emtricitabine; tenofovir disoproxil	3,221	73.15 (70.51, 75.89)	67.87 (65.58, 70.23)	201,564	6.01 (5.93)	64.44 (62.12)
Zoledronic acid	1,415	4.65 (4.41, 4.91)	4.63 (4.40, 4.88)	3,946.28	2.19 (2.11)	4.55 (4.32)
Adefovir	718	85.72 (79.37, 92.58)	78.27 (72.95, 83.97)	54,205.6	6.27 (6.02)	77.39 (71.65)
Medroxyprogesterone	640	11.39 (10.53, 12.32)	11.26 (10.42, 12.16)	5,926.96	3.48 (3.34)	11.15 (10.31)
Emtricitabine; rilpivirine; tenofovir disoproxil	572	60.69 (55.74, 66.08)	56.87 (52.51, 61.59)	31,145.7	5.82 (5.56)	56.36 (51.77)
Pamidronic acid	570	8.97 (8.25, 9.74)	8.89 (8.19, 9.65)	3,958.74	3.14 (3.00)	8.82 (8.12)
Anastrozole	484	7.71 (7.05, 8.44)	7.65 (7.00, 8.37)	2,780.71	2.93 (2.77)	7.60 (6.95)
Cobicistat; elvitegravir; emtricitabine; tenofovir disoproxil	457	48.51 (44.14, 53.32)	46.04 (42.10, 50.36)	20,014.3	5.51 (5.24)	45.72 (41.59)
Prednisolone	416	3.42 (3.11, 3.77)	3.41 (3.10, 3.76)	705.3	1.76 (1.61)	3.40 (3.08)
Omeprazole	377	2.40 (2.17, 2.66)	2.40 (2.17, 2.66)	306.36	1.26 (1.10)	2.39 (2.16)
Letrozole	287	3.12 (2.77, 3.50)	3.11 (2.77, 3.49)	409.11	1.63 (1.45)	3.10 (2.76)
Lamivudine	230	5.28 (4.64, 6.01)	5.26 (4.62, 5.98)	790.68	2.39 (2.17)	5.24 (4.60)
Raloxifene	169	7.38 (6.35, 8.59)	7.33 (6.31, 8.52)	922.68	2.87 (2.60)	7.31 (6.29)
Phenytoin	163	2.66 (2.28, 3.10)	2.65 (2.28, 3.09)	167.8	1.41 (1.17)	2.65 (2.27)
Exemestane	107	4.08 (3.37, 4.93)	4.06 (3.36, 4.91)	247.06	2.02 (1.70)	4.06 (3.36)
Asfotase alfa	93	3.38 (2.75, 4.14)	3.37 (2.75, 4.12)	154.64	1.75 (1.42)	3.36 (2.74)
Alendronic acid; colecalciferol	86	12.79 (10.34, 15.83)	12.63 (10.23, 15.58)	920.37	3.66 (3.16)	12.61 (10.19)
Lopinavir; ritonavir	82	3.31 (2.66, 4.11)	3.30 (2.66, 4.09)	131.29	1.72 (1.36)	3.30 (2.65)
Cobicistat; elvitegravir; emtricitabine; tenofovir	49	22.50 (16.94, 29.88)	21.96 (16.65, 28.96)	980.66	4.46 (3.54)	21.94 (16.52)
Darunavir	41	3.70 (2.72, 5.03)	3.69 (2.72, 5.00)	80.31	1.88 (1.35)	3.68 (2.71)
Calcitriol	40	6.86 (5.03, 9.36)	6.81 (5.00, 9.28)	198.57	2.77 (2.12)	6.81 (4.99)
Triptorelin	40	3.34 (2.45, 4.56)	3.33 (2.45, 4.54)	65.39	1.74 (1.20)	3.33 (2.44)
Calcitonin	35	7.80 (5.59, 10.88)	7.74 (5.57, 10.77)	205.56	2.95 (2.22)	7.74 (5.55)
Deflazacort	27	3.41 (2.34, 4.98)	3.40 (2.34, 4.96)	45.89	1.77 (1.10)	3.40 (2.33)
Emtricitabine; tenofovir	22	4.37 (2.88, 6.65)	4.36 (2.87, 6.61)	56.96	2.12 (1.32)	4.36 (2.87)
Magnesium sulfate	20	5.81 (3.74, 9.01)	5.77 (3.73, 8.94)	79.01	2.53 (1.60)	5.77 (3.72)
Tenofovir alafenamide	17	3.36 (2.09, 5.42)	3.35 (2.09, 5.39)	28.11	1.75 (0.89)	3.35 (2.08)
Efavirenz; lamivudine; tenofovir disoproxil	11	30.22 (16.57, 55.13)	29.25 (16.36, 52.30)	300.42	4.87 (2.28)	29.25 (16.03)
Emtricitabine	11	5.07 (2.80, 9.17)	5.05 (2.80, 9.10)	35.71	2.33 (1.08)	5.04 (2.79)

**TABLE 2:** Time-to-onset analysis of shared positive drug signals using the Weibull distribution.

​			Weibull distribution				​
​	Cases	TTO (days)	Scale parameter		Shape parameter		​
Drug name	n	Median (IQR)	α	95% CI	β	95% CI	Failure type
Alendronic acid; colecalciferol	22	375.50 (90.00, 707.00)	812.96	459.12–1,439.50	0.86	0.61–1.21	Random failure
Anastrozole	127	304.00 (31.00, 731.00)	553	443.73–689.18	0.92	0.79–1.08	Random failure
Asfotase alfa	9	488.00 (127.00, 1386.00)	704.06	307.32–1,612.96	0.83	0.49–1.41	Random failure
Calcitonin	4	318.00 (90.50, 1272.50)	931.58	300.49–2,888.12	1.06	0.44–2.57	Random failure
Calcitriol	4	786.00 (0.00, 1747.00)	1826.82	1,616.04–2065.09	11.94	3.76–37.89	Wear-out failure
Cobicistat; elvitegravir; emtricitabine; tenofovir	3	187.00 (126.00, 370.00)	258.49	156.58–426.73	2.39	0.99–5.79	Random failure
Cobicistat; elvitegravir; emtricitabine; tenofovir disoproxil	77	876.00 (274.00, 1854.00)	1,190.2	956.00–1,481.77	1.08	0.90–1.30	Random failure
Darunavir	14	580.50 (228.00, 1253.00)	780.27	387.79–1,569.94	0.81	0.51–1.27	Random failure
Deflazacort	5	577.00 (115.00, 651.00)	573.4	193.39–1700.11	0.85	0.41–1.74	Random failure
Efavirenz; emtricitabine; tenofovir disoproxil	486	1879.50 (834.00, 3005.00)	2,248.38	2,103.64–2,403.08	1.4	1.31–1.51	Wear-out failure
Emtricitabine	2	1,026.00 (309.00, 1743.00)	1,125.78	391.94–3,233.58	1.39	0.44–4.40	Random failure
Emtricitabine; rilpivirine; tenofovir disoproxil	81	871.00 (410.00, 1865.00)	1,319.85	1,097.41–1,587.37	1.25	1.05–1.49	Wear-out failure
Emtricitabine; tenofovir	4	1,016.00 (406.00, 3308.50)	1,699.82	499.99–5,778.88	0.85	0.39–1.83	Random failure
Emtricitabine; tenofovir disoproxil	876	1,606.00 (730.00, 3034.50)	2,182.51	2064.84–2,306.87	1.26	1.20–1.33	Wear-out failure
Esomeprazole	173	1,326.00 (519.00, 2200.00)	1756.68	1,564.40–1972.58	1.39	1.23–1.57	Wear-out failure
Exemestane	23	245.00 (59.00, 554.00)	577.15	339.45–981.29	0.89	0.63–1.26	Random failure
Lamivudine	12	2,399.50 (880.50, 4109.00)	2,871.01	2073.16–3,975.90	1.91	1.16–3.14	Wear-out failure
Letrozole	92	276.50 (14.50, 698.00)	454.7	332.54–621.72	0.73	0.61–0.87	Early failure
Lopinavir; ritonavir	23	947.00 (163.00, 2194.00)	1,159.98	684.16–1966.71	0.81	0.59–1.13	Random failure
Medroxyprogesterone	341	2,922.00 (1803.00, 3782.00)	3,238.78	3,065.89–3,421.42	2.05	1.88–2.23	Wear-out failure
Omeprazole	36	2,283.00 (303.50, 4257.00)	2,754.71	1863.17–4,072.87	0.9	0.67–1.21	Random failure
Pamidronic acid	108	926.00 (293.00, 1763.50)	1,240.54	1,015.67–1,515.19	1	0.86–1.17	Random failure
Phenytoin	21	6,148.00 (3,287.00, 10,957.00)	8,702.44	5,824.51–13,002.4	1.16	0.79–1.70	Random failure
Prednisolone	27	214.00 (5.00, 821.00)	556.91	342.01–906.84	0.92	0.65–1.30	Random failure
Raloxifene	34	1,033.50 (306.00, 2191.00)	1,629.27	1,143.98–2,320.42	1.06	0.79–1.42	Random failure
Tenofovir disoproxil	445	2047.00 (808.00, 3753.00)	2,586.48	2,394.14–2,794.28	1.27	1.18–1.37	Wear-out failure
Triptorelin	15	0.00 (0.00, 1081.00)	1,122.1	729.85–1725.18	1.8	0.95–3.41	Random failure
Zoledronic acid	300	396.00 (122.00, 798.00)	573.5	497.39–661.26	0.86	0.78–0.94	Early failure

### 3.6 Comprehensive evidence assessment and causal plausibility evaluation

Based on the modified Bradford-Hill criteria, a multidimensional qualitative assessment of causal plausibility was conducted for the 33 high-confidence drug signals. In terms of signal strength, ROR values for TDF and its combinations, adefovir, and bisphosphonates were substantially higher than those for other drugs, indicating more pronounced reporting disproportionality (Table 3). In terms of consistency, FAERS and VigiAccess showed high concordance in signal direction and drug categories, and the results were stable across gender and age subgroups. In terms of specificity, bisphosphonates were primarily associated with osteonecrosis and fractures, whereas nucleoside (acid) analogues were primarily associated with osteomalacia and hypophosphatemic lesions, suggesting distinct phenotypic patterns with different mechanisms. Temporal analysis showed that tenofovir, glucocorticoids, and proton pump inhibitors (PPIs) were associated with wear-out failure events (β > 1, delayed TTO), whereas bisphosphonates and aromatase inhibitors were mostly associated with early-failure events (β < 1), consistent with their pharmacological characteristics. In terms of biological plausibility, the observed signal patterns were consistent with known pharmacological mechanisms reported in the literature. For example, tenofovir and adefovir have been associated with renal tubular phosphate wasting and impaired bone mineralization; glucocorticoids are known to suppress osteogenesis and enhance bone resorption; aromatase inhibitors and progestins may reduce estrogen-mediated bone protection; and bisphosphonates, despite their antiresorptive role, have been linked to hypocalcemia and osteonecrosis in specific clinical settings. Comparative analysis further showed that drugs within similar therapeutic classes tended to exhibit comparable signal patterns. Taken together, these findings support robust pharmacovigilance signals and substantial causal plausibility for associations between tenofovir, bisphosphonates, PPIs, glucocorticoids, aromatase inhibitors, and metabolic bone disorders.

**TABLE 3:** Bradford–Hill-based qualitative assessment of causal plausibility for major drug classes associated with metabolic bone disorders (MBDs).

Criterion	Qualitative interpretation	Data source/Method
Strength of the association	In both databases (FAERS and VigiAccess), five major drug classes showed consistent positive pharmacovigilance signals, with ROR values ranging from 12.72 to 85.72. The strongest signals were observed for nucleos(t)ide analogues (e.g., adefovir, tenofovir disoproxil, and emtricitabine/tenofovir-containing combinations), followed by bisphosphonates (e.g., alendronate and zoledronic acid) and aromatase inhibitors (e.g., anastrozole and letrozole). Concordant signal positivity across the four disproportionality algorithms (ROR, PRR, BCPNN, and MGPS) supported the strength of reporting disproportionality for these drug–event associations	Disproportionality analysis using four algorithms (ROR, PRR, BCPNN, MGPS)
Consistency	Signal patterns were highly consistent between FAERS and VigiAccess, and remained stable across gender, age, and reporter type subgroups. Positive controls (e.g., bisphosphonates) and negative controls (e.g., calcium preparations) exhibited expected signal directions, supporting the consistency of the observed pharmacovigilance signals across data sources and subgroup analyses	Cross-validation between FAERS and VigiAccess; stratified analyses by gender, age, and reporter type
Specificity	Different drug classes were associated with distinct MBD-related reporting phenotypes. Tenofovir and adefovir were more frequently linked to hypophosphatemic osteopathy, whereas bisphosphonates were more commonly associated with osteonecrosis and atypical fractures, and aromatase inhibitors with estrogen deficiency-related osteoporosis. These phenotype-specific reporting patterns were considered supportive of specificity within the modified Bradford–Hill framework	Based on MedDRA PT-level phenotype mapping and ATC-based drug classification
Temporality	Weibull distribution analysis suggested different time-to-onset reporting patterns across drug classes. Nucleos(t)ide analogues, glucocorticoids, and PPIs more often showed wear-out failure patterns (β > 1), suggesting that reports may become more frequent with longer treatment duration, whereas bisphosphonates and aromatase inhibitors more often showed early-failure patterns (β < 1), suggesting that reports may occur more frequently during earlier treatment periods. These temporal patterns should be interpreted cautiously, particularly where drug-specific report counts were limited	Time-to-onset (TTO) and Weibull shape parameter analysis based on FAERS data
Biological plausibility/Empirical evidence	The observed signal patterns were broadly consistent with known pharmacological mechanisms reported in the literature. For example, tenofovir and adefovir have been associated with renal tubular phosphate wasting and impaired bone mineralization; glucocorticoids are known to suppress osteoblast activity and enhance bone resorption; PPIs may reduce calcium absorption; and aromatase inhibitors and progestins may decrease estrogen-mediated bone protection. These findings were considered supportive of biological plausibility	Literature-informed pharmacological interpretation
Coherence	The findings of this study are consistent with previous evidence on drug-induced bone loss and osteomalacia. Prior case reports and clinical trials confirmed similar adverse skeletal outcomes within the same drug classes, strengthening biological coherence	Literature validation and comparison with prior pharmacovigilance studies
Analogy	Similar bone metabolism impairments were observed among drugs of the same pharmacological class (e.g., various tenofovir analogues, different bisphosphonates), suggesting a class effect that supports analogical reasoning	Comparative analysis with drugs of similar structure or mechanism

Abbreviations: FAERS, U.S. Food and Drug Administration Adverse Event Reporting System; ROR, Reporting Odds Ratio; PRR, Proportional Reporting Ratio; BCPNN, Bayesian Confidence Propagation Neural Network; MGPS, Multi-item Gamma–Poisson Shrinker; MedDRA, Medical Dictionary for Regulatory Activities; ATC, Anatomical Therapeutic Chemical classification system; TTO, Time to Onset; β, Weibull shape parameter; MBD, Metabolic Bone Disease.

## 4 Discussion

This study systematically analyzed AE reports related to drug-associated MBD using the FAERS and the World Health Organization global database, VigiAccess. Using four disproportionality algorithms, 42 drugs with significant signals were identified. Further cross-database integration revealed that 33 of these drugs exhibited consistent positive signals in both FAERS and VigiAccess, supporting the robustness and cross-database consistency of the observed pharmacovigilance signals. Based on a multidimensional Bradford-Hill-based qualitative assessment, these drug signals showed support across multiple evaluated dimensions, including strength, consistency, specificity, temporality, and biological plausibility, supporting the causal plausibility of signal-based associations between these drugs and metabolic bone disorders. First, in terms of signal strength and consistency, bisphosphonates (e.g., alendronic acid, zoledronic acid), nucleoside (acid) analogues (e.g., TDF, adefovir, emtricitabine combination), aromatase inhibitors (e.g., anastrozole, letrozole), and proton pump inhibitors (e.g., esomeprazole) showed strong and highly consistent positive signals in both databases, with corresponding ROR values ranging from 12.7 to 85.7, indicating marked reporting disproportionality across both databases. To provide an additional descriptive interpretation of signal magnitude, IC025 values have been categorized in previous pharmacovigilance studies as weak (0 <IC025 ≤ 1.5), moderate (1.5 < IC025 ≤ 3.0), and strong (IC025 > 3.0). However, because disproportionality metrics arise from different statistical frameworks and no universally accepted cutoff exists across all settings, this categorization was considered descriptive only and was not used as the primary basis for signal evaluation in the present study (Mikaeili et al., 2025; Yang H. et al., 2022). Second, in phenotype-specific analyses, different drug classes exhibited distinct patterns of bone metabolism injury: nucleoside (acid) analogues were more frequently associated with hypophosphatemic osteomalacia, bisphosphonates were more commonly linked to osteonecrosis and atypical fractures, aromatase inhibitors and progestins were associated with estrogen deficiency-related osteoporosis, and glucocorticoids were linked to secondary osteoporosis, consistent with existing reports (Black et al., 2019; Zhang et al., 2025; Chakrabarti and McCune, 2023; Yang X. et al., 2022). TTO and Weibull distribution analysis suggested distinct temporal reporting patterns across different drug classes. Bisphosphonates and aromatase inhibitors more often showed an early-failure pattern (β < 1), suggesting that reported events may occur more frequently during the earlier treatment period. In contrast, nucleos(t)ide analogues, glucocorticoids, and PPIs more often showed a wear-out failure pattern (β > 1), suggesting that reported events may become more frequent with longer treatment duration. Overall, these temporal patterns were broadly consistent with previous clinical observations and with biological plausibility. For example, long-term tenofovir exposure has been associated with renal tubular phosphate loss and impaired bone mineralization, chronic glucocorticoid use has been linked to disruption of bone remodeling balance (Zhu et al., 2025; Paccou et al., 2024), and long-term acid suppression therapy may reduce calcium absorption (Freedberg et al., 2017). In the sex- and age-stratified analyses, reports from female patients were more frequent than those from male patients (62.49% vs. 30.32%), which may reflect sex-specific susceptibility, differences in drug exposure patterns, or reporting behavior. This pattern may be particularly relevant in postmenopausal women, who have increased baseline vulnerability to bone metabolic abnormalities. This result is closely related to bone metabolic fragility caused by decreased estrogen levels (Compston et al., 2019). Reports from the elderly group were primarily associated with bisphosphonates and anticancer drugs, whereas those from the younger group were primarily related to antiviral drugs and glucocorticoids, consistent with the drug exposure characteristics of different age groups (Saag et al., 1998; Sølling et al., 2025). Analysis of reporting sources revealed that healthcare professionals were more concerned with the known risks associated with osteoporosis drugs and antiviral drugs, whereas reports from consumers covered a wider range of drug classes, such as PPIs and hormonal drugs, suggesting that public awareness of the potential bone risks of long-term medication use is increasing.

Cross-database signal integration and Bradford-Hill-based qualitative assessment further supported the robustness and consistency of the observed pharmacovigilance signals. The five major drug classes (bisphosphonates, nucleos(t)ide analogues, glucocorticoids, aromatase inhibitors, and PPIs) showed consistent signal patterns, and the Bradford-Hill-based evaluation supported the causal plausibility of their associations with metabolic bone disorders across the assessed dimensions, including strength, consistency, specificity, and biological plausibility. Among these, tenofovir and its combination regimens showed the greatest signal magnitude (ROR >60), suggesting that monitoring of bone health may be warranted during long-term antiviral treatment (Hadzimuratovic et al., 2021; Joseph et al., 2023). Although alendronate and zoledronic acid remain mainstays of osteoporosis treatment, their long-term use has also been associated with osteonecrosis and atypical fractures, highlighting the need to balance therapeutic benefit against potential bone-related adverse outcomes. Aromatase inhibitors and progestins have likewise been associated with reduced estrogen-mediated bone protection, underscoring the importance of bone health monitoring during endocrine therapy. The signals observed for PPIs were also consistent with epidemiological evidence suggesting an association between long-term PPI exposure and increased fracture risk.

Mechanistically, the observed signal patterns were broadly consistent with known pharmacological mechanisms. Tenofovir and adefovir have been associated with renal tubular phosphate wasting and impaired bone mineralization (Yang X. et al., 2022); glucocorticoids are known to suppress osteoblast differentiation, promote osteocyte apoptosis, and enhance osteoclast activity (Paccou et al., 2024); aromatase inhibitors reduce estrogen synthesis and disrupt bone remodeling balance (Zhang et al., 2025); long-term PPI use may reduce calcium absorption through acid suppression (Freedberg et al., 2017); and bisphosphonates, despite their antiresorptive role, have been linked to hypocalcemia, osteonecrosis, and impaired bone remodeling in specific clinical settings (Sølling et al., 2025). The consistency between these known mechanisms and the observed signal patterns further supports the biological plausibility of these drug-event associations.

The present findings may provide useful evidence for clinical drug safety awareness and bone health surveillance. For patients receiving long-term treatment with glucocorticoids, aromatase inhibitors, antiviral agents, or PPIs, bone density assessment and monitoring of bone metabolism may be considered, particularly in individuals at elevated baseline risk (Grossmann et al., 2019). For patients treated with bisphosphonates over prolonged periods, periodic reassessment of treatment duration or consideration of drug holidays may be appropriate in selected clinical settings to reduce the risk of atypical fractures and osteonecrosis (Watts et al., 2021). In addition, for patients undergoing long-term antiviral treatment for HIV or hepatitis B, switching from TDF to tenofovir alafenamide (TAF) may be considered where clinically appropriate to reduce potential bone toxicity (Buti et al., 2016; Pan et al., 2024).

Although this study is systematic and broadly representative, several limitations should be acknowledged. First, reporting bias and underreporting inherent to spontaneous reporting systems may lead to incomplete or distorted signal estimates. Second, the observational nature of these databases limits direct causal inference, and important clinical variables, such as dose, treatment duration, comorbidities, and concomitant medications, were not consistently available. Third, some drug-specific temporal estimates, particularly in Weibull analyses, may be unstable when based on limited report counts and should therefore be interpreted cautiously. Fourth, because most reports in FAERS and VigiAccess originate from Europe and North America, the geographic generalizability of the findings requires further evaluation. Future studies integrating electronic health records, prospective cohorts, and mechanistic multi-omics data may help refine these pharmacovigilance signals and further clarify the biological basis of drug-associated metabolic bone disorders.

## 5 Conclusion

This study systematically analyzed drug risk signals associated with MBD using two global pharmacovigilance databases, FAERS and VigiAccess. Using four disproportionality algorithms (ROR, PRR, BCPNN, and MGPS), robust pharmacovigilance signals and disproportionality-based associations involving 33 drugs and MBD were identified. A comprehensive Bradford Hill-based assessment further supported the causal plausibility of the observed disproportionality-based associations between bisphosphonates, glucocorticoids, aromatase inhibitors, antiviral drugs, proton pump inhibitors, and metabolic bone disorders. This dual-database analysis provides high-confidence real-world evidence for pharmacovigilance signal detection in drug-associated metabolic bone abnormalities and offers valuable insights for clinical drug safety and bone health management.

## Data Availability

The original contributions presented in the study are included in the article/Supplementary Material, further inquiries can be directed to the corresponding author.
